# Corrigendum to “Characterization of the glutathione redox state in the Golgi apparatus” [Redox Biol. 81 (2025) 103560]

**DOI:** 10.1016/j.redox.2025.103652

**Published:** 2025-05-03

**Authors:** Carla Miró-Vinyals, Sarah Emmert, Gina Grammbitter, Alex Jud, Tobias Kockmann, Pablo Rivera-Fuentes

**Affiliations:** a Department of Chemistry, University of Zurich, Zurich, Switzerland; b Institute of Chemical Sciences and Engineering, École Polytechnique Fédérale de Lausanne, Lausanne, Switzerland; c Department of Biochemistry, University of Zurich, Zurich, Switzerland; d Functional Genomics Center Zurich, ETH Zurich/University of Zurich, Zurich, Switzerland

The authors regret that the original version of the paper cited only one of the two proteomics datasets available on the PRIDE platform to support this paper. The two datasets have the following accession codes: PXD060873 and PXD060874.

The authors would like to apologise for any inconvenience caused.

