# Supplementary material for: Characterization of the glutathione redox state in the Golgi apparatus
Source: Redox Biol. 2025 Feb 19;81:103560. doi: 10.1016/j.redox.2025.103560 (PMC11904595; doi:10.1016/j.redox.2025.103560)
Supplement: Multimedia component 1 [file mmc1.pdf]

Supporting Information

For manuscript:

Characterization of the Glutathione Redox State in the Golgi Apparatus

Carla Miró-Vinyals,[1] Sarah Emmert,[1] Gina Grammbitter,[2] Alex Jud,[3] Tobias Kockmann,[4] Pablo Rivera-Fuentes[1]*

1. Department of Chemistry, University of Zurich, Zurich, Switzerland.
2. Institute of Chemical Sciences and Engineering, École Polytechnique Fédéral de Lausanne, Lausanne, Switzerland.
3. Department of Biochemistry, University of Zurich, Zurich, Switzerland.
4. Functional Genomics Center Zurich, ETH Zurich/University of Zurich, Zurich, Switzerland.

* Corresponding author: pablo.riverafuentes@uzh.ch

1. Supplementary tables

Table S1. List of antibodies.

Name and host	Conjugated	Supplier	Supplier number
Mouse anti-HA	No	ThermoFisher	26183
Rabbit anti-GM130	No	ThermoFisher	PA5-95727
Goat anti-mouse	Alexa 488	ThermoFisher	A32723
Donkey anti-rabbit	Alexa 680	abcam	ab186692
Mouse-anti-GAPDH	Dylight 488	ThermoFisher	MA5-15738-D488
Rabbit-anti-HA	No	ThermoFisher	PA1-985

Table S2. Microscope settings for imaging channels.

Channel	λ excitation	Emission filter
roGFP blue	405 nm	525/50
roGFP green	445 nm	525/50
mGold	515 nm	542/27
TRaQ-G	561 nm	642 LP
Alexa Fluor 488	488 nm	525/50
Alexa Fluor 680	561 nm	642 LP

Table S3. Plasmid sources

Plasmid	Source	Vector	Insert
Calnexin-Halo-mGold	Addgene 183989	-	-
pmTurquoise2-Golgi	Addgene 36205	-	-
B4GALT1-Grx1-roGFP1-iE	TwistBioscience	-	-
The B4GALT1-Grx1-roGFP1-iE gene sequence is available in Addgene cloned into a pCMV backbone (to be added)			
HA-sCGrx1p-6His	TwistBioscience	-	-
sCGrx1pER	A gift from Appenzeller-Herzog[16]	-	-
B4GALT1-HA-sCGrx1p	Gibson assembly	B4GALT1-pmTurquoise2	sCGrx1pER[16]
B4GALT1-sCGrx1p-HA	Gibson assembly (Addgene -to be added)	B4GALT1-HA-sCGrx1p	B4GALT1-HA-sCGrx1p and B4GALT1-HA-sCGrx1p
HA-B4GALT1-sCGrx1p	Gibson assembly	B4GALT1-HA-sCGrx1p	B4GALT1-HA-sCGrx1p

			B4GALT1-HA-sCGrx1p
HT7-mGold-6His	Addgene 183986		
B4GALT1-HT7-mGold	Gibson assembly (Addgene -to be added)	B4GALT1-pmTurquoise2	Calnexin-Halo-mGold

Table S4. Primers for Gibson assembly

Plasmid	Fragments	Primers	
B4GALT1-HA-sCGrx1p	Vector: B4GALT1-pmTurquoise2	For	TTGCAAATtaaagcggccgcgact
		Rev	ATACGGATAcaccatggtggcgacc
	Insert: SCGRX1P-er	For	accatggtgTATCCGTATGATGTGCCTGACTACGC
		Rev	gccgctttaATTTGCAAGAATAGGTTCTAACAATTCCTCC
HA-B4GALT1-sCGrx1p	Vector: B4GALT1-ha-scgrx1p	For	cgccaccGTATCTCAAGAAACTATCAAGCACGTCAAG
		Rev	ATACGGATAcatggtggcgagct
	Insert 1: galt1-HA-scgrx1p	For	gccaccatgTATCCGTATGATGTGCCTGAC
		Rev	ccgaagcctTTCTTGAGATACCATGGTACCTTCTGC
	Insert 2: galt1-ha-SCGRX1P	For	TCAAGAAaggcttcgggagccg
		Rev	TGAGATACggtggcgaccggtgg
B4GALT1-sCGrx1p-HA	Vector: B4GALT1-ha-scgrx1p	For	ACGCAGAAGGTACCtaaagcggcc
		Rev	GTACCTTCggtggcgaccggtggat
	Insert 1: galt1-HA-scgrx1p	For	TGCAAATgtcgccaccatggtgTATCC
		Rev	gccgctttaGGTACCTTCTGCGTAGTCAGG
	Insert 2: galt1-ha-SCGRX1P	For	cgccaccGAAGGTACCATGGTATCTCAAGAAACTATCAAG
		Rev	gtggcgacATTTGCAAGAATAGGTTCTAACAATTCCTCC
B4GALT1-HT7-mGold	pmTurquoise2-Golgi	For	aagtaaagcggccgc
		Rev	atttcggtggcgacc
	Calnexin-Halo-mGold	For	cgccaccgaaatcggtact
		Rev	cggccgctttacttgtacag

Table S5: Important sequences of used GOIs for mammalian cell expression.

Plasmid	GOI sequence
B4GALT1	aggcttcgggagccgctcctgagcggcagcgccgcgatgccaggcgcgtccctacagcgggcctgccgcct<br>gctcgtggccgtctgcgctctgcaccttggcgtcaccctcgtttactacctggctggccgcgacctgagccgcctg<br>ccccaactggtcggagtctccacaccgctgcag
Grx1-roGFP2	atggctcaagagtttgtgaactgcaaaatccagcctgggaaggtggttgtgttcatcaagcccacctgcccgtac<br>tgcaggagggcccaagagatcctcagtcaattgcccatcaaacaagggcttctggaatttgtcgatatcacagc<br>caccaaccacactaacgagattcaagattatttgcaacagctcacgggagcaagaacggtgcctcgagtcttt<br>attggtaaagattgtataggcggatgcagtgatctagtctctttgcaacagagtggggaactgctgacgcggcta<br>aagcagattggagctctgcagactagtggtggttcaggtggtggtggttcaggtggtggtggttcaggtggagga<br>ggatcaggaggaggaggatcaggaggaggaggatcaggaggagaattcgtgagcaagggcgaggagct<br>gttcaccggggtggtgcccatcctggtcgagctggacggcgacgtaaacggccacaagttcagcgtgtccggc<br>gagggcgagggcgatgccacctacggcaagctgaccctgaagttcatctccaccaccggcaagctgcccgt<br>gccctggcccaccctcgtgaccaccctgacctacggcgtgcagtgcttcagccgctaccccgaccacatgaag<br>cagcacgacttcttcaagtccgccatgcccgaaggctacgtccaggagcgcaccatcttcttcaaggacgacg<br>gcaactacaagacccgcgccgaggtgaagttcgagggcgacaccctggtgaaccgcatcgagctgaaggg<br>catcgacttcaaggaggacggcaacatcctggggcacaagctggagtacaactacaactgccacaacgtct<br>atatcatggccgacaagcagaagaacggcatcaaggtgaacttcaagatccgccacaacatcgaggacgg<br>cagcgtgcagctcgccgaccactaccagcagaacacccccatcggcgacggccccgtgctgctgcccgaca<br>accactacctgagcacctgctccgccctgagcaaagaccccaacgagaagcgcgatcacatggtcctgctgg<br>agttcgtgaccgccgccgggatcactctcggcatggacgagctgtacaag
Grx1-<br>roGFP1-iE	atggctcaagagtttgtgaactgcaaaatccagcctgggaaggtggttgtgttcatcaagcccacctgcccgtac<br>tgcaggagggcccaagagatcctcagtcaattgcccatcaaacaagggcttctggaatttgtcgatatcacagc<br>caccaaccacactaacgagattcaagattatttgcaacagctcacgggagcaagaacggtgcctcgagtcttt<br>attggtaaagattgtataggcggatgcagtgatctagtctctttgcaacagagtggggaactgctgacgcggcta<br>aagcagattggagctctgcagactagtggtggttcaggtggtggtggctcgggtggtggtggatcaggaggag<br>gcggtagtggaggaggtggaagcggcggaggaggatcaggaggagaattcgtgagcaagggcgaggag<br>ctgttcaccggggtggtgcccatcctggtcgagctggacggcgacgtaaacggccacaagttcagcgtgtccg<br>gcgagggcgagggcgatgccacctacggcaagctgaccctgaagttcatctccaccaccggcaagctgccc<br>gtgccctggcccaccctcgtgaccaccctgagctacggcgtgcagtgcttcagccgctaccccgaccacatga<br>agcagcacgacttcttcaagtccgccatgcccgaaggctacgtccaggagcgcaccatcagcttcaaggacg<br>acggcaactacaagacccgcgccgaggtgaagttcgagggcgacaccctggtgaaccgcatcgagctgaa<br>gggcatcgacttcaaggaggacggcaacatcctggggcacaagctggagtacaactacaactgcgagagc<br>aacgtctatatcaccgccgacaagcagaagaacggcatcaaggctaacttcaagacccgccacaacatcga<br>ggacggcagcgtgcagctcgccgaccactaccagcagaacacccccatcggcgacggccccgtgctgctg<br>cccgacaaccactacctgagcacctgctccgccctgagcaaagaccccaacgagaagcgcgatcacatggt<br>cctgctggagttcgtgaccgccgccgggatcactctcggcatggacgagctgtacataagctaa
sCGrx1p-HA	atggtatctcaagaaactatcaagcacgtcaaggaccttattgcagaaaacgagatcttcgtcgcatccaaaac<br>gtactgtccatactctcatgcagctttaaacacgctttttgaaaagttaaaggttcccaggtccaaagttctggttttg<br>caattgaatgacatgaaggaaggcgcagacattcaggctgcgttatatgagattaatggccaaagaaccgtgc<br>caaacatctatattaatggtaaacatattggaggcaacgacgacttgcaggaattgagggagactggtgaattg<br>gaggaattgttagaacctattcttgcaaatgtcgccaccatggtgtatccgtatgatgtgcctgactacgca
HT-mGold	gaaatcggtactggctttccattcgacccccattatgtggaagtcctgggcgagcgcatgcactacgtcgatgttg<br>gtccgcgcgatggcacccctgtgctgttcctgcacggtaacccgacctcctcctacgtgtggcgcaacatcatcc<br>cgcatgttgcaccgacccatcgctgcattgctccagacctgatcggtatgggcaaatccgacaaaccagacct<br>gggttatttcttcgacgaccacgtccgcttcatggatgccttcatcgaagccctgggtctggaagaggtcgtcctg<br>gtcattcacgactggggctccgctctgggtttccactgggccaagcgcaatccagagcgcgtcaaaggtattgc<br>atttatggagttcatccgccctatcccgacctgggacgaatggccagaatttgcccgcgagaccttccaggcctt<br>ccgcaccaccgacgtcggccgcaagctgatcatcgatcagaacgtttttatcgagggtacgctgccgatgggtg<br>tcgtccgcccgctgactgaagtcgagatggaccattaccgcgagccgttcctgaatcctgttgaccgcgagcca

	ctgtggcgcttcccaaacgagctgccaatcgccggtgagccagcgaacatcgtcgcgctggtcgaagaatac<br>atggactggctgcaccagtcccctgtcccgaagctgctgttctggggcaccccaggcgttctgatcccaccggc<br>cgaagccgctcgcctggccaaaagcctgcctaactgcaaggctgtggacatcggcccgggtctgaatctgctg<br>caagaagacaacccggacctgatcggcagcgagatcgcgcgctggctgtccacgctcgagatttccggcgtg<br>agcaagggcgaggagctgttcaccggggtggtgcccatcctggtcgagctggacggcgacgtaaacggcca<br>caagttcagcgtgtccggcgagggcgagggcgatgccacctacggcaagctgaccctgaagttcatctgcac<br>caccggcaagctgcccgtgccctggcccaccctcgtgaccagcctgggctacggcctgcagtgcttcgcccgc<br>taccccgaccacatgaagcagcacgacttcttcaagtccgccatgcccgaaggctacgtccaggagcgcacc<br>atcttcttcaaggacgacggcaactacaagacccgcgccgaggtgaagttcgagggcgacaccctggtgaa<br>ccgcatcgagctgaagggcatcgacttcaaggaggacggcaacatcctggggcacaagctggagtacaact<br>acaacagccacaacgtctatatcaccgccgacaagcagaagaacggcatcaaggccaacttcaagatccg<br>ccacaacatcgaggacggcggcgtgcagctcgccgaccactaccagcagaacacccccatcggcgacgg<br>ccccgtgctgctgcccgacaaccactacctgagctaccagtccaagctgagcaaagaccccaacgagaagc<br>gcgatcacatggtcctgctggagttcgtgaccgccgccgggatcactctcggcatggacgagctgtacaag

2. Supplementary figures

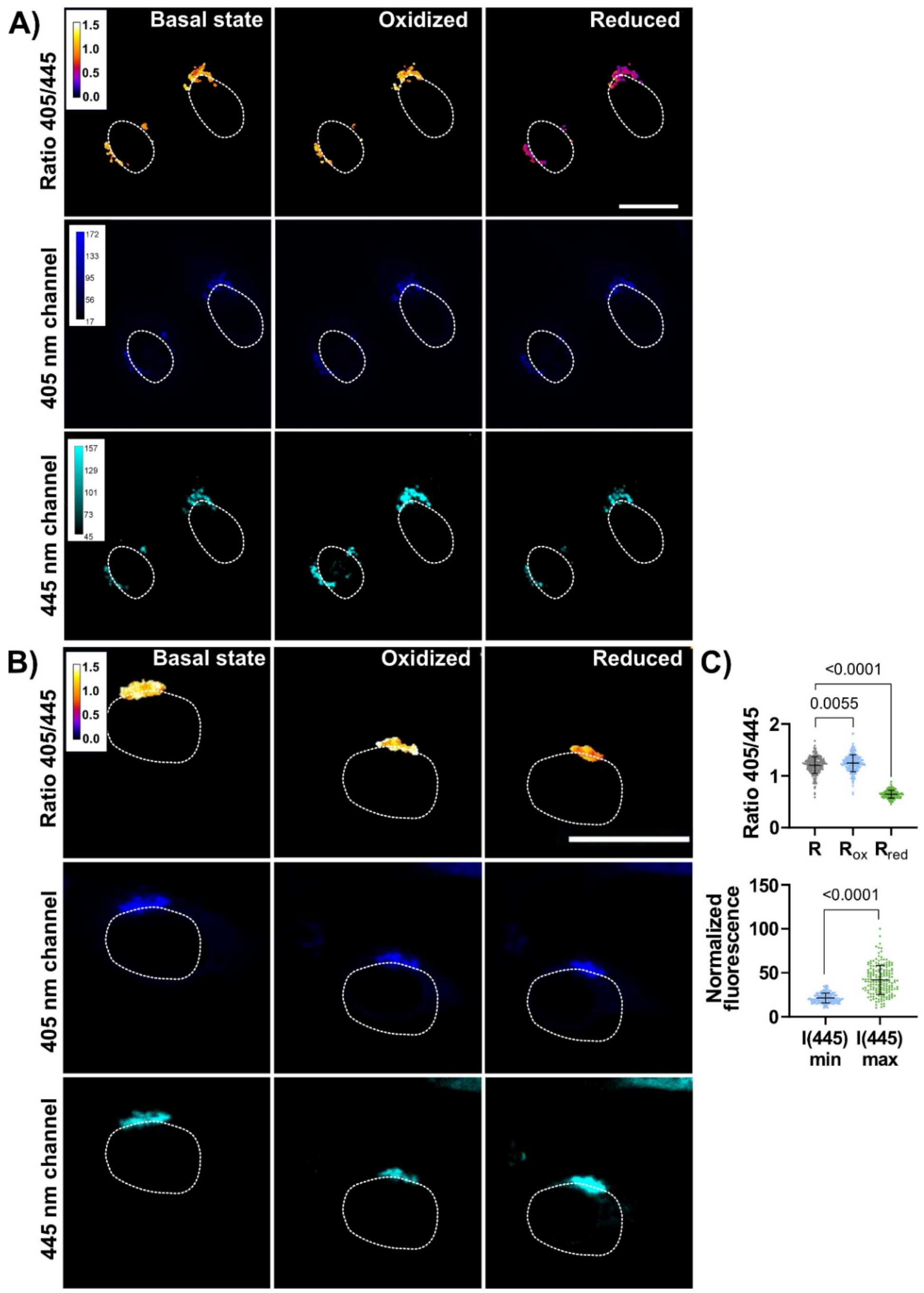

Figure S1. HeLa **(A)** and HEK293 (**B** and **C**) cells transiently transfected with B4GALT1-Grx1-roGFP1-iE. **A** and **B)** 405 nm channel, 445 nm channel, and ratiometric fluorescent images with excitation at 405 nm and 445 nm wavelength and emission at 525/50 nm. The cells were imaged at basal state conditions, under oxidizing conditions (after 3 min of adding 1 mM H_2O_2), and under reducing conditions (after 3 min of adding 5 mM DTT). Scale bar = 20 µm. C) Data analysis of n ≥ 50, technical replicates, average of three biological replicates. Statistical significance was assessed by unpaired, two-tailed, Mann-Whitney test.

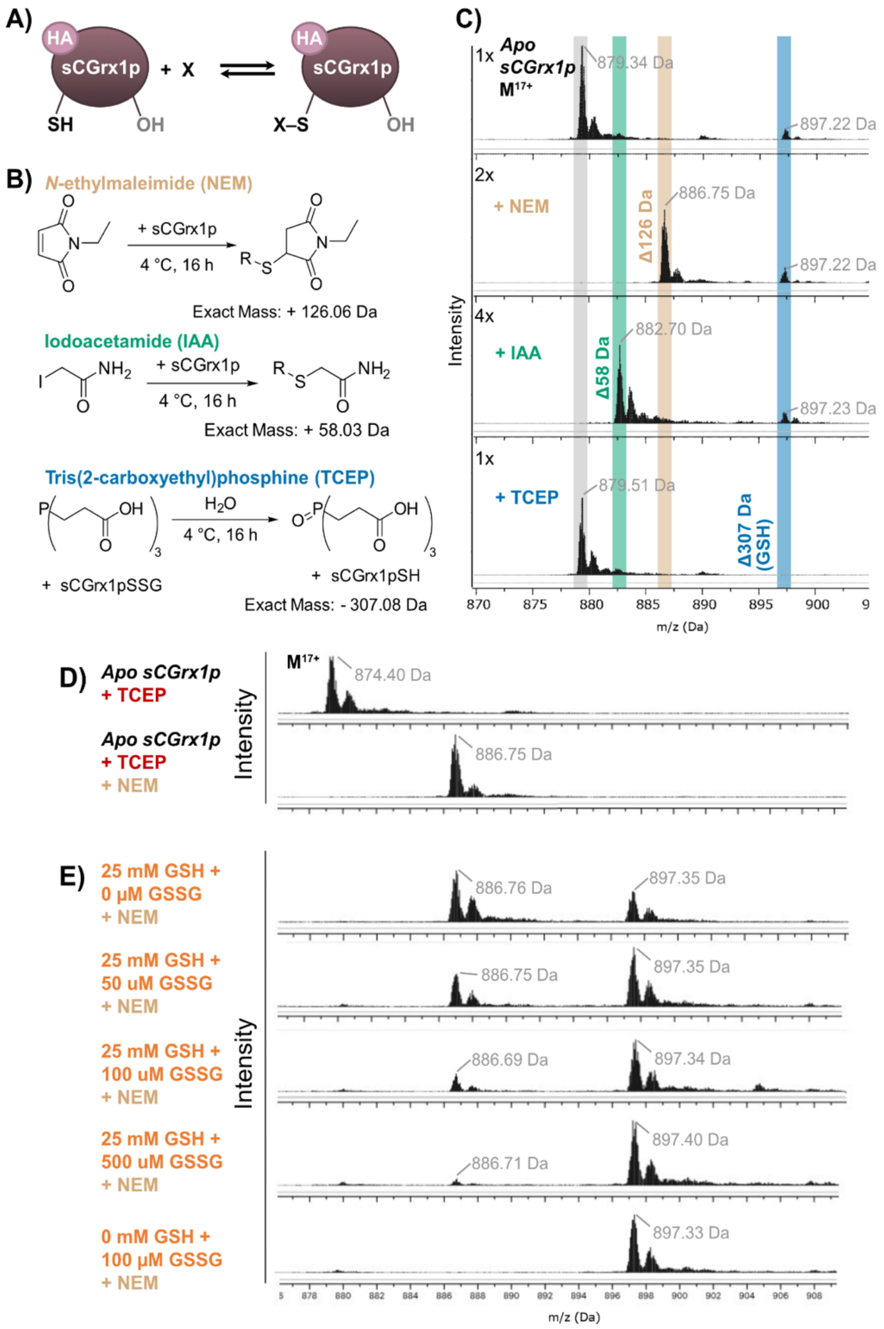

Figure S2. sCGrx1p *in vitro* alkylation assays analyzed via protein MS. **A)** Schematic reaction of cysteine alkylation. **B)** Reaction scheme of sCGrx1p alkylation with NEM and IAA, and reduction with TCEP. **C)** HR-ESI-UPLC-MS zoomed-in spectra of the different samples with indications of the expected mass changes upon chemical modification. All the reactions were performed in PBS (100 µM protein and 2 mM reagent for 4 °C, 16 h). **D)** HR-ESI-MS zoomed in spectra of the apo-protein samples (100 µM) with prereduction with TCEP (0.5 mM, storage buffer) and NEM reaction (50 mM, 4 °C, 16 h). **E)** HR-ESI-MS zoomed-in spectra of protein samples incubated with GSH and increasing amounts of GSSG. The protein (100 µM) was pre-reduced with TCEP (0.5 mM, storage buffer), and the buffer was exchanged to PBS pH 7.2. The reduced protein was reacted with GSH/GSSG (20 °C, 10 min) and immediately capped by reaction with NEM (50 mM, 4 °C, 16 h).

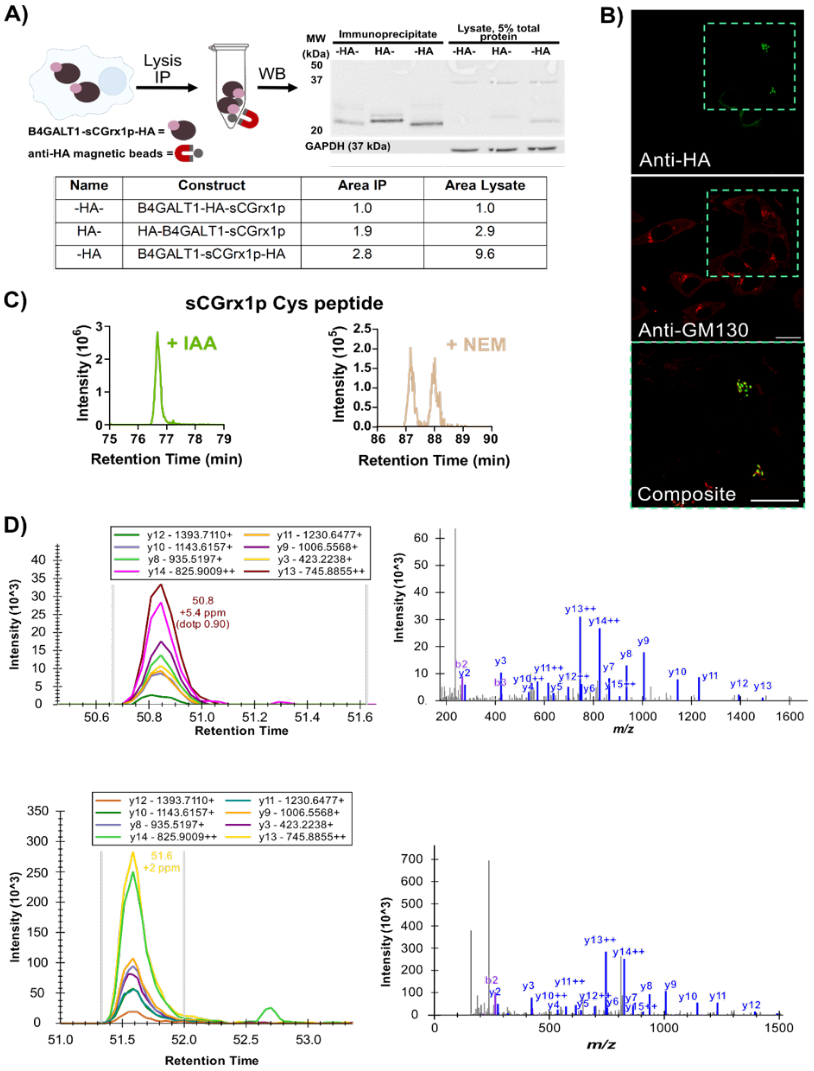

Figure S3. A) Schematic IP procedure and western blot analysis of the HA-tagged constructs transiently expressed in HeLa cells. Shown are the immunoprecipitates (after incubation at 4 °C for 16 h) from anti-HA magnetic beads (left) and raw lysate samples containing 5% of total protein (right). Bottom, Quantification of HA availability for HA IP. GAPDH was used as a control for overall expression levels. Areas were measured with Fiji and normalized regarding the -HA- construct. An additional normalization was carried out to the expression of GAPDH. **B)** Fluorescence microscopy images of fixed HeLa cells transiently expressing B4GALT1-sCGrx1p-HA. Mouse anti-HA and anti-mouse-Alexa488 antibodies were used to label the construct, and rabbit anti-GM130 and anti-rabbit-Alexa680 were used to label the Golgi marker GM130. **C)** Extracted ion chromatogram (XIC) traces in MS1 of the sCGrx1p peptide containing the reactive cysteine and the alkylating modification (TYCPYSHAALNTLFEK, 638.9733 Da [M]+++ for IAA, 661.6487 Da [M]+++ for NEM). **D)** Fragment ion traces in Skyline for HeLa (top) and HEK293 (bottom) and MS/MS spectra of the peptide containing the modification. Y14++ was chosen as the quantification fragment since it is the most abundant fragment containing the IAA modification.

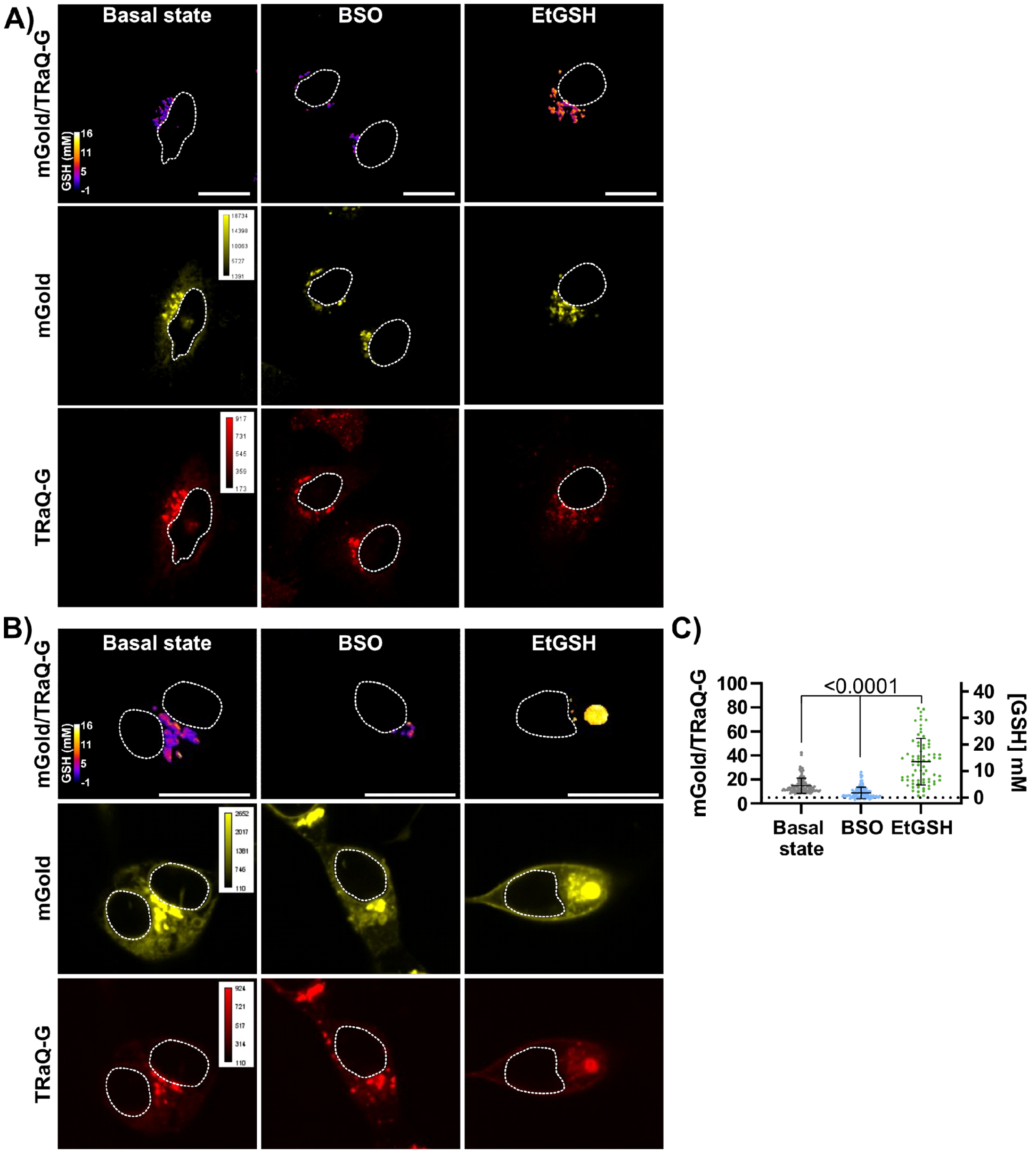

Figure S4. HeLa (A) and HEK293 (B and C) cells transiently transfected with B4GALT1-HT-mGold and incubated with 100 nM TRaQ-G ligand for 1 h at 37 °C. **A)** and **B)** mGold channel, TRaQ-G channel, and mGold/TRaQ-G ratiometric images of HeLa (A) and HEK293 (B) cells. Excitation at 515 nm and emission at 542/27 nm for mGold, and excitation at 561 nm and emission at 642 long-pass filter for TRaQ-G. The color calibration is illustrated in concentration values, not ratios. The cells were imaged at basal state conditions, under BSO treatment (1 mM, 3–4h incubation) and under EtGSH conditions (10 mM, 3–4h incubation). Scale bar = 20 µm. C) Data analysis of n ≥ 50, technical replicates (individual cells), average of three biological replicates. Statistical significance was assessed by unpaired, two-tailed, Mann-Whitney test.
